# CVD incidence and mortality among people with diabetes and/or hypertension: Results from the English longitudinal study of ageing

**DOI:** 10.1371/journal.pone.0303306

**Published:** 2024-05-31

**Authors:** Paola Zaninotto, Andrew Steptoe, Eun-Jung Shim

**Affiliations:** 1 Department of Epidemiology and Public Health, UCL, London, United Kingdom; 2 Department of Behavioural Science and Health, UCL, London, United Kingdom; 3 Department of Psychology, Pusan National University, Busan, Republic of Korea; University of Debrecen, HUNGARY

## Abstract

**Background and aims:**

Diabetes and/or hypertension are the most common conditions in older people, and also related to higher cardiovascular disease (CVD) incidence and mortality. This study aims to explore the risk of CVD incidence and mortality among older people with diabetes and/or hypertension over a 16 years follow-up period and investigates the role of depression and obesity in these relationships.

**Methods:**

6,855 participants aged 50+ from the English Longitudinal Study of Ageing (ELSA). The main exposure is having diabetes and/or hypertension at baseline (2002/2003) compared to not having, but excluded those with coronary heart disease (CHD) and/or stroke (CVD). Survival models are used for CVD incidence and mortality up to 2018, adjusted for socio-demographic, health, health behaviours, cognitive function, and physical function characteristics.

**Results:**

39.3% of people at baseline had diabetes and/or hypertension. The risk of CVD incidence was 1.7 (95%CI: 1.5; 1.9) higher among people with diabetes and/or hypertension compared to those without and was independent of covariates adjustment. People with diabetes and/or hypertension were also 1.3 (95%CI: 1.1; 1.8) times more likely to die from CVD than those without. We did not find evidence for an elevated risk of CVD incidence and mortality among people with obesity nor among those with depression.

**Conclusions:**

In order to effectively reduce the risk of CVD incidence and mortality among older people, treatment as well as management of hypertension and diabetes should be routinely considered for older people with diabetes and/or hypertension.

## Introduction

Among the non-communicable diseases, cardiovascular disease (CVD), including stroke, is one of the leading contributor to the global burden of disease [1]. CVD include angina and myocardial infarction (also known as ischemic heart disease-IHD), and other heart conditions. Between 2009 and 2019 in the UK a decline in IHD morbidity has been observed [2]. Nevertheless, ischemic heart disease and stroke were the top two causes of deaths, respectively, in the UK for the same period [2]. However, the CVD burden comes also from living with the disease, and as the population of older persons in many countries continues to grow the burden is set to increase and pose more challenges to the health systems.

CVD incidence and mortality are much higher among people diabetes and/or hypertension [3–7]. The incidence of both diabetes and hypertension increases with age and are therefore most common in older people [8]. Hypertension is widely recognized as the most significant modifiable risk factor for CVDs [9], and a leading contributor of disability-adjusted life years [10]. The global prevalence of diabetes is predicted to increase [6] posing significant challenges to health systems. Diabetes is related to substantial medical expenditures [11] as well as with increased mortality [12] and CVD morbidity [13]. Between 1994 and 2017 the prevalence of diabetes among people aged 65 and older increased from 5.6% to 15% in the United Kingdom [14].

The Whitehall II study, a cohort study with civil servants in the UK, observed that not only depression predicted coronary heart disease (CHD), but there was also a dose-response effect in that higher frequency of GHQ-30 caseness was related to a greater risk of CHD incidence over the last 5-year follow up, implying a causal relationship between depression and CHD. Depressive symptoms were also predictor of stroke events over 5 year cycles in this study [15].

Association of depression have been reported with a greater risk of non-fatal and fatal CVD among individuals with type 2 diabetes [16]. A meta-analysis observed a significant positive association between depression and risk of stroke morbidity and mortality, and this association diminished after adjusting for smoking or BMI (suggesting that these factors may be mediating factors of the association) [17].

In a prospective study of representative sample of adults from the Health Survey for England and Scottish Health Survey (1994–2004), both hypertension and common mental disorder (i.e., GHQ-12 defined caseness) were independently associated with an elevated risk of CVD and all-cause mortality. The highest risk of CVD mortality was observed in individuals with combined diagnosis of hypertension and common mental disorder [18].

In the current study we assess CVD incidence and mortality over a period of 16 years in a large sample of community dwellers older people with diabetes and/or hypertension. We contribute the existing literature in three novel ways: 1) we take into account a wider range of covariates than previous work, including measures of cognitive and physical function; 2) we use competing risk analysis methods which allows to account for loss to follow-up and alternative causes of death more effectively than in previous work; 3) we explore first the effect modification role of depression and then the effect modification role obesity in the relationship between diabetes and/or hypertension and CVD incidence and mortality. Several studies have shown that obesity and depression are common among people with diabetes/hypertension [19–23] and are also risk factors for CVD events [16,24–27]. We therefore hypothesise that people with diabetes and/or hypertension who are also depressed have higher risk of CVD incidence and mortality than those with diabetes and/or hypertension but who are not depressed. Similarly, we hypothesize that people with diabetes and/or hypertension who are also obese have higher risk of CVD incidence and mortality than those with diabetes and/or hypertension but who are not obese. Lastly, we separately evaluate the impact of each condition on CVD outcomes, as well as the coexistence of the two, within our sample.

## Materials and methods

### Data

The data are from the English Longitudinal Study of Ageing (ELSA) [28], which began in 2002-2003(first phase of data collection referred to as Wave 1), a nationally representative sample of individuals aged 50 and older living in private households in England, followed and re-interviewed every 2 years. The main objective of the study is to understand the complex dynamics of the ageing process, that is, the relationships between economic and family circumstances, behaviour, social participation, biology, retirement, and health and well-being [29]. Data collection comprises of face-to-face interviews, self-completion questionnaires and nurse visits in participants’ homes every other wave [29]. The analytical sample for this work included 6,855 people who at baseline had diabetes and/or hypertension compared to those without, but excluded those with CVD and/or stroke. The study complies with the Declaration of Helsinki. All ELSA participants provided written consent prior to the study, and ethical approval was granted by the London Multi-Centre Research Ethics Committee. Data are made available through the UK Data Service.

#### Exposure: Diabetes and/or hypertension

At each study interview participants were asked whether a doctor or nurse told them they had any of the following health conditions: angina, myocardial infarction, stroke, any other heart condition (including heart failure, heart murmur, arrhythmia), diabetes, hypertension, mental conditions (depression, anxiety, mood swings, emotional problems), psychiatry conditions (psychosis, schizophrenia, hallucinations, bipolar disorder), respiratory illness, arthritis, cancer, dementia (including Parkinson’s disease and Alzheimer’s disease) and eye-related condition (glaucoma, diabetic eye, macular degeneration). From answers to these questions we computed and indicator to define people with diabetes and/or hypertension (156 with diabetes, 2146 with hypertension, and 270 with diabetes and hypertension) at baseline but excluding those with CVD and stroke at baseline (2,354 and 510 respectively), from this sample we further excluded people that at baseline did not have hypertension or diabetes but developed it during the follow-up (958 cases).

#### Incident CVD outcome

From the baseline interview up to wave 8 (2016–2017) there were a total of 1,233 CVD and stroke events (of which 300 of stroke).

#### Mortality outcome

Study participants were linked to the National Health Service’s Central Registry which provide vital status data. For each deceased participant, the month and year of death are recorded up to the end of follow-up (April 2018). Also, data regarding causes of death are provided for broad classifications of disease according to the *International Classification of Diseases*. These classifications also included cardiovascular disease and stroke. During the follow-up period, there were a total of 1655 deaths, of which 555 were from CVD and stroke (in the whole sample). Follow-up began on the date of study induction (2002 to 2003) with study members censored at date of death or end of follow-up (May 2018).

#### Covariates

Socio-demographic factors included age (ranging from 50 to 96), gender, ethnicity (white and non-white), cohabitation status (currently living or not with a partner whether married or not), educational attainment (medium/high (A-levels, college and above) vs low (below O-levels)), working status (in paid employment vs not in paid employment), wealth tertiles (high, medium and low). Mental and physical health factors included depressive symptoms (3+ or more out of the 8 symptoms collected from the Centre for Epidemiological Study Scale depression scale, 8-items), the presence of a limiting longstanding illness, self-rated health (excellent, very good and good vs fair poor) and comorbidities (1 or more chronic conditions). Health behavioural factors included physical activity (physically active vs inactive/sedentary), frequency of alcohol consumption (less than daily vs daily), and obesity categories (computed from objectively measured BMI at either wave 0 or wave 2). Cognitive function variables included memory, assessed using a word-list learning test (total score ranged from 0 to 20 with higher scores indicating better cognitive function) and executive function assessed through the animal naming test (ranging from 4 to 24, higher scores indicating more animals named in one minute). Physical functioning variables included mobility items, and limitations with activity and instrumental activities of daily living (none vs 1 or more).

### Statistical analyses

Descriptive statistics were adjusted for the survey design and weighted for non-response. Chi-square tests and t-tests were used to assess for statistical significance between those with diabetes/hypertension and those without. To examine the association between diabetes/hypertension and incident CVD we employed competing—risk regression analysis with subdistribution hazard ratios (SHR) and related 95% Confidence Intervals, using a version of the Fine and Gray method [30]. This method allows a competing risk—an event that might occur during the follow-up instead of the event of interest—to also be taken into account in the analysis. In this case death (1,665 cases) and study drop-out (2,266 cases) are potential competing risks when examining incidence rates of CVD-stroke, therefore it was important to take this into account rather than treating those who had died or dropped out as censored. CVD deaths were also included as competing risks, as in our sample people are only interviewed every two years, therefore they might have died of CVD before we could record their diagnosis. Having ascertained that the proportional hazards assumption had not been violated, we used the Cox models to compute hazard ratios with accompanying 95% confidence intervals to summarise the relationship between diabetes/hypertension and CVD deaths. Several adjustment models were used for incidence CVD and mortality according to adjustment: age and sex; age, sex and sociodemographic characteristics; age, sex and health characteristics; age, sex and behavioural factors; age, sex and cognitive function; age, sex and physical functioning; and lastly fully adjusted models. The analytical sample for this work included 6,855 people who at baseline had diabetes and/or hypertension (N = 2572) compared to those without, but excluded those with CVD and/or stroke and those with missing data on covariates (687 cases, 9.1%).

To further assess the combined and individual effects of hypertension and diabetes on CVD risk and mortality, we repeated the fully adjusted models using as exposure a 4-factor variable indicating whether people have: diabetes only (N = 156), hypertension only (N = 2146), both (N = 270) and neither (N = 4127).

Lastly, we run two further analyses as follows:

1. To explore whether people with diabetes/hypertension who also report having depression are at higher risk of CVD incidence and mortality, compared to those who have diabetes/hypertension but who are not depressed, we tested whether depression was an effect modifier by adding an interactions term between the main exposure and depression in the fully adjusted models.
2. To explore whether people with diabetes/hypertension who also report being obesity are at higher risk of CVD incidence and mortality, compared to those who have diabetes/hypertension but who are not obese, we tested whether obesity was an effect modifier by adding an interaction term between the main exposure and obesity in the fully adjusted models.

### Sensitivity analysis

The first sensitivity analysis included exploring the relationship between diabetes/hypertension with incidence CHD and stroke separately.

## Results

In Table 1, we report baseline characteristics of study participants according to diabetes/hypertension status. With the exception of ethnicity, education, smoking and alcohol consumption, people with diabetes/hypertension reported worse profiles than those without. For example, on average they were older, had lower cognitive function scores, were more likely to not cohabiting with a partner, not to be in paid employment, to be in the poorest group of wealth, to be depressed, to have a limiting longstanding illness, a higher prevalence of co-morbidities, and poor self-rated health. Furthermore, they were also more likely to be physically inactive, to have lower cognitive function and poorer physical functioning. Incidence CVD was higher among people with diabetes/hypertension than those without (24% vs 14%); furthermore, the prevalence of CVD deaths among people with diabetes/hypertension was almost double of that of people without diabetes/hypertension (11% vs 6%).

**Table 1:** Sample characteristics at baseline, England 2002–2003.

	No	Diabetes/Hypertension	p-value
n	4283	2572	
%	60.7	39.3	<0.001
Female	53.9	56.0	0.093
Mean age	62.0	64.4	<0.001
S.D.	9.4	9.6	
Non-white ethnicity	2.2	2.8	0.108
Not cohabiting with a partner	28.0	32.1	0.004
Low education	51.5	53.0	0.243
Not in paid work	47.6	60.7	<0.001
Richest wealth	34.4	30.4	<0.001
Medium wealth	33.5	33.2	
Poorest wealth	32.1	36.4	
Depressed	19.0	24.2	<0.001
1+ Chronic conditions	45.5	54.3	<0.001
fair/poor SRH	15.6	25.8	<0.001
Limiting Longstanding illness	23.1	31.9	<0.001
Daily alcohol consumption	29.3	28.2	0.320
Never smoked	36.9	37.0	<0.001
Ex-smoker	41.4	47.9	
Current smoker	21.7	15.1	
Physically inactive	12.4	17.0	<0.001
Normal BMI	36.1	19.1	<0.001
Overweight	43.6	43.6	
Obese	20.2	37.3	
Memory CF	17.1	16.3	<0.001
S.D.	4.9	4.9	
Executive CF (animal naming)	17.8	17.2	<0.001
S.D.	4.2	4.2	
**Physical function**			
1+ ADLs	13.3	18.6	<0.001
1+ IADLs	13.8	18.4	<0.001
1+ Mobility	45.4	58.5	<0.001
CVD events N = 1,033	13.6	24.0	<0.001
CHD events N = 933	11.6	19.8	<0.001
Stroke Events N = 300	2.8	6.6	<0.001
CHD or stroke deaths N = 555	6.3	11.1	<0.001
Other deaths N = 1,061	22.1	25.1	

Notes. Estimates adjusted for the complex survey design and weighted for non-response sampling weights.

Events and deaths are reported for the entire follow-up period.

In Table 2, we report the subdistribution hazard ratios (SHR) and related 95% Confidence Intervals (CI) for the association between diabetes and/or hypertension and CVD incidence, according to adjustment variables. We can see that people with diabetes and/or hypertension were more likely to develop CVD incidence than those without, this was true according to all adjustment models. In the full model the SHR was 1.66 (95%CI 1.48; 1.88) compared to 1.75 of the age and sex adjusted model only (1.56; 1.96), suggesting that this relationship was not fully explained nor attenuated by covariates. The results also showed that other than the main exposure, age and gender (female) have minimal impact on CVD incidence. Non-white ethnicity and socioeconomic factors like not cohabiting and not being in paid work show varied risks. Conditions like a limiting longstanding illness and chronic disease slightly increase the risk of CVD incidence, while lifestyle factors like physical inactivity and obesity also show some association with increased risk (which then disappear in the fully adjusted model). The average time at risk was 14 years (min 2.0 year and maximum 15.3 years) among those with no diabetes and/or hypertension and 13.5 years (min 1.9 year and maximum 15.3 years).

**Table 2:** Subdistribution hazard ratios and 95% confidence intervals for incident CVD among people with diabetes and/or hypertension compared to people without.

	SHR (95%CI)	SHR (95%CI)	SHR (95%CI)	SHR (95%CI)	SHR (95%CI)	SHR (95%CI)	SHR (95%CI)
**Diabetes/Hypertension**	**1.8 (1.6; 2.0)**	**1.7 (1.5; 1.9)**	**1.7 (1.5; 1.9)**	**1.7 (1.5; 1.9)**	**1.8 (1.6; 2.0)**	**1.7 (1.5; 1.9)**	**1.7 (1.5; 1.9)**
Age	1.0 (1.0; 1.0)	1.0 (1.0; 1.0)	1.0 (1.0; 1.0)	1.0 (1.0; 1.0)	1.0 (1.0; 1.0)	1.0 (1.0; 1.0)	1.0 (1.0; 1.0)
Female	0.9 (0.8; 1.0)	0.9 (0.8; 1.0)	0.9 (0.8; 1.0)	0.9 (0.8; 1.0)	0.9 (0.8; 1.0)	0.9 (0.8; 1.0)	0.9 (0.8; 1.0)
Non-white ethnicity		0.5 (0.3; 0.8)					0.6 (0.3; 0.9)
Not cohabiting with a partner		1.1 (1.0; 1.3)					1.1 (0.9; 1.2)
Low education		1.0 (0.9; 1.2)					1.0 (0.9; 1.2)
Not in paid work		1.2 (1.0; 1.4)					1.2 (1.0; 1.3)
Medium wealth		1.1 (1.0; 1.3)					1.1 (1.0; 1.3)
Poorest wealth		1.1 (0.9; 1.2)					1.1 (0.9; 1.2)
Depressed			1.2 (1.1; 1.4)				1.2 (1.1; 1.4)
1+ Chronic conditions			1.1 (1.0; 1.3)				1.1 (1.0; 1.3)
Fair/poor SRH			1.0 (0.9; 1.2)				1.0 (0.8; 1.2)
Limiting Longstanding illness			1.2 (1.0; 1.3)				1.0 (0.9; 1.2)
Daily alcohol consumption				1.0 (0.9; 1.1)			1.0 (0.9; 1.1)
Current smoker				1.0 (0.9; 1.1)			1.0 (0.9; 1.1)
Physically inactive				1.1 (1.0; 1.3)			1.0 (1.0; 1.2)
Overweight				1.0 (0.8; 1.1)			1.0 (0.8; 1.1)
Obese				1.1 (1.0; 1.3)			1.0 (0.9; 1.2)
Memory CF					1.0 (1.0; 1.1)		1.0 (1.0; 1.1)
Executive CF (animal naming)					1.0 (1.0; 1.1)		1.0 (1.0; 1.1)
1+ ADLs						1.3 (1.1; 1.5)	1.2 (1.0; 1.4)
1+ IADLs						1.0 (0.9; 1.2)	1.0 (0.8; 1.2)
1+ Mobility						1.4 (1.2; 1.5)	1.3 (1.1; 1.5)

Note. 544 events in people with diabetes/hypertension and 489 in those without.

**Fig 1:**
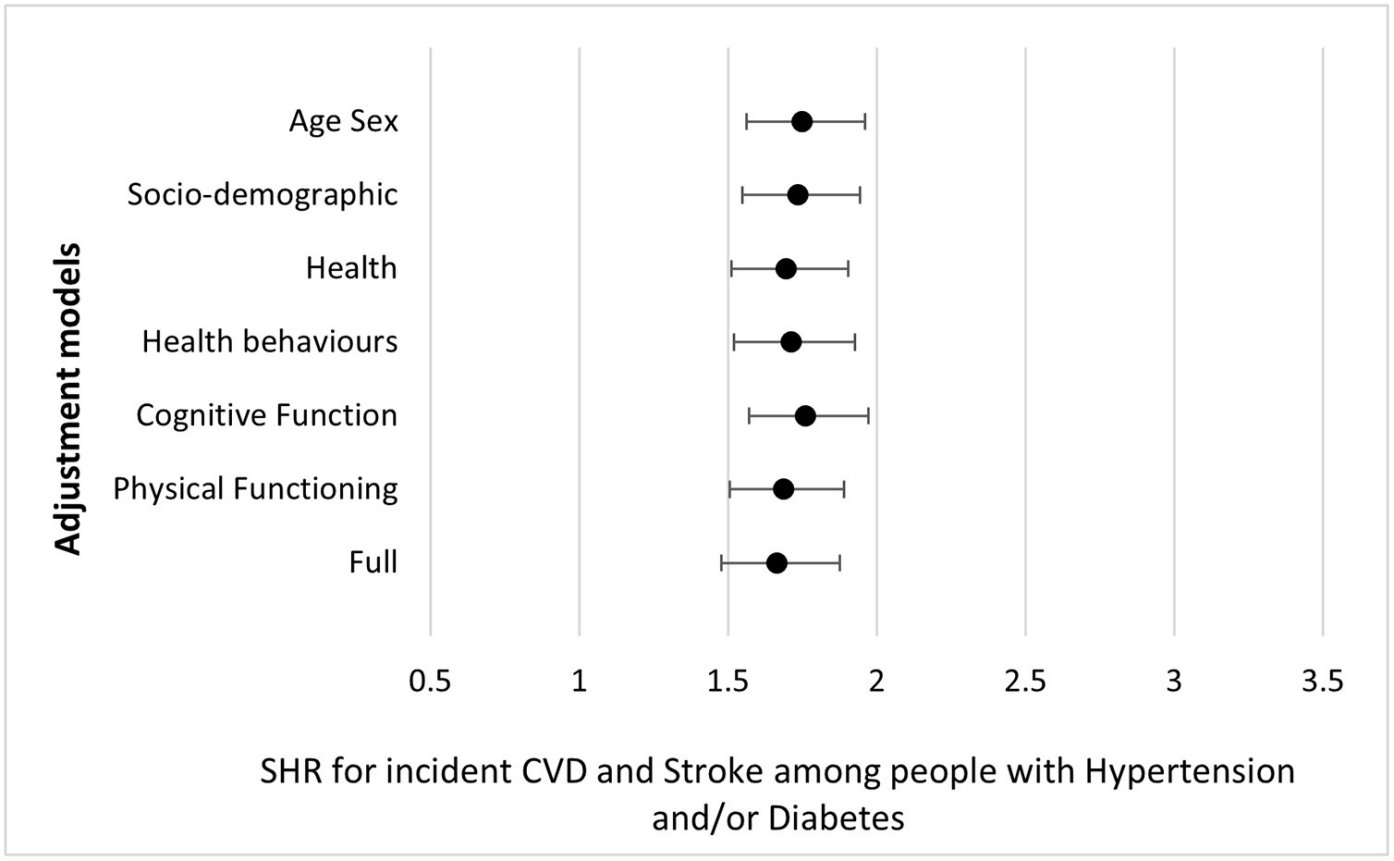
Subdistribution Hazard Ratios and 95%Confidence Intervals for incident CVD among people with diabetes and/or hypertension compared to people without.

In Fig 1, we show graphically the results of Table 2 for the different models and for the full model.

Table 3 shows the Hazard Ratios (HR) for CVD mortality in people with diabetes/hypertension compared to those without. We can see that in the model adjusted for age and sex only, people with diabetes/hypertension were 1.5 times more likely to die of CVD than those without (HR 1.5 95%CI 1.26; 1.77). In the fully adjusted model the HR decreased to 1.31 (95%CI 1.10; 1.56), but remained statistically significant. Factors associated with the risk of CVD mortality, after accounting for hypertension and or/ diabetes include age, gender (being female appears to be protective), non-white ethnicity, not cohabiting with a partner, lower education levels, not being in paid work, wealth status (with poorer wealth associated with higher risk), depression, presence of chronic conditions, self-rated health (fair/poor being higher risk), and lifestyle factors such as physical inactivity, smoking, and being overweight or obese. Cognitive and functional abilities also play a role, as indicated by measures like memory cognitive function and physical functioning. The average time at risk was 13.9 years (min 2.0 year and maximum 15.3 years) among those with no diabetes and/or hypertension and 13.2 years (min 1.9 year and maximum 15.3 years).

**Table 3:** Hazard ratios and 95% confidence intervals for CVD mortality among people with diabetes and/or hypertension compared to people without.

	HR (95%CI)	HR (95%CI)	HR (95%CI)	HR (95%CI)	HR (95%CI)	HR (95%CI)	HR (95%CI)
**Diabetes/Hypertension**	**1.5 (1.3; 1.8)**	**1.4 (1.2; 1.7)**	**1.4 (1.2; 1.6)**	**1.5 (1.2; 1.7)**	**1.5 (1.2; 1.7)**	**1.4 (1.2; 1.7)**	**1.3 (1.1; 1.8)**
Age	1.1 (1.1; 1.1)	1.1 (1.1; 1.1)	1.1 (1.1; 1.1)	1.1 (1.1; 1.1)	1.1 (1.1; 1.1)	1.1 (1.1; 1.1)	1.1 (1.1; 1.1)
Female	0.6 (0.5; 0.7)	0.6 (0.5; 0.7)	0.6 (0.5; 0.7)	0.6 (0.5; 0.7)	0.6 (0.5; 0.7)	0.6 (0.5; 0.7)	0.6 (0.5; 0.7)
Non-white ethnicity		0.5 (0.2; 1.0)					0.4 (0.2; 0.8)
Not cohabiting with a partner		1.4 (1.1; 1.7)					1.4 (1.2; 1.7)
Low education		0.9 (0.8; 1.1)					0.8 (0.7; 1.0)
Not in paid work		1.9 (1.5; 2.4)					1.7 (1.3; 2.1)
Medium wealth		1.6 (1.2; 2.0)					1.4 (1.1; 1.8)
Poorest wealth		2.1 (1.6; 2.6)					1.6 (1.2; 2.0)
Depressed			1.0 (0.8; 1.2)				0.8 (0.6; 1.0)
1+ Chronic conditions			1.0 (0.9; 1.2)				1.0 (0.9; 1.2)
Fair/poor SRH			1.8 (1.5; 2.3)				1.5 (1.2; 1.9)
Limiting Longstanding illness			1.2 (0.9; 1.4)				1.1 (0.8; 1.3)
Daily alcohol consumption				1.0 (0.8; 1.2)			1.1 (0.9; 1.3)
Current smoker				1.3 (1.2; 1.5)			1.2 (1.1; 1.4)
Physically inactive				1.9 (1.5; 2.3)			1.4 (1.1; 1.7)
Overweight				0.9 (0.7; 1.1)			0.9 (0.7; 1.1)
Obese				1.1 (0.9; 1.4)			1.1 (0.9; 1.4)
Memory CF					1.1 (0.9; 1.0)		1.0 (1.0; 1.0)
Executive CF (animal naming)					0.9 (0.9; 1.0)		1.0 (0.9; 1.0)
1+ ADLs						1.1 (0.9; 1.4)	0.9 (0.7; 1.1)
1+ IADLs						1.4 (1.1; 1.7)	1.0 (0.8; 1.3)
1+ Mobility						1.3 (1.1; 1.6)	1.1 (0.9; 1.4)

Note. 297 CVD deaths in people with diabetes/hypertension and 258 in those without.

**Fig 2:**
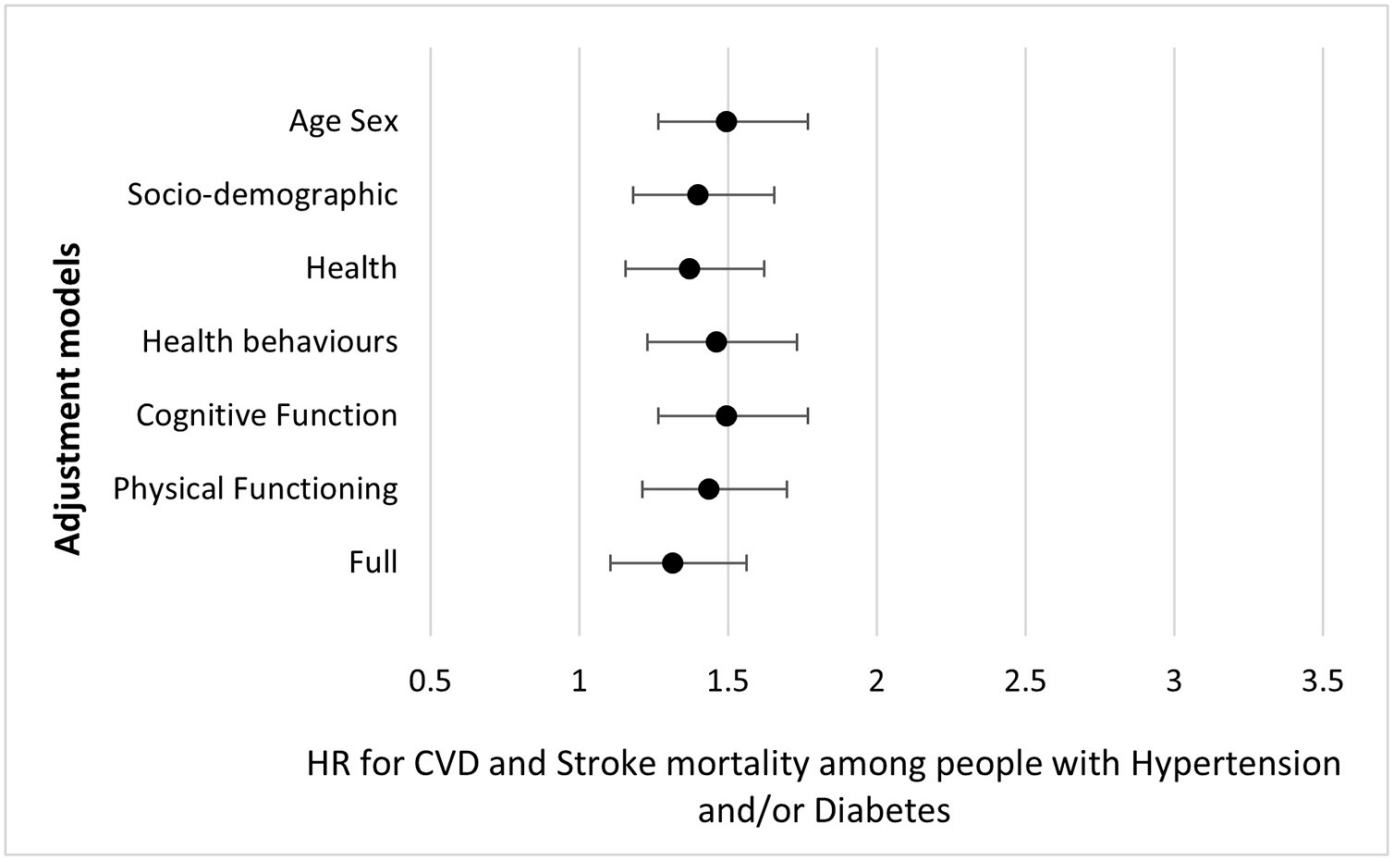
Hazard Ratios and 95% confidence intervals for CVD mortality among people with diabetes and/or hypertension compared to people without.

In Fig 2, we show the HR and corresponding confidence intervals for the different models and for the full model.

### The role of depression

Depression was significantly associated with higher risk of CVD incidence (Table 2) in both the age, sex and health adjusted model (SHR:1.23 95%CI:1.07;1.42) and the fully adjusted models (SHR:1.22 95%CI:1.05;1.41) over and above diabetes/hypertension (Table 2). However, we did not find evidence for depression being an effect modifier in this relationship (SHR 1.01 p-value = 0.111, 95%CI 0.60; 1.05), meaning that people with diabetes/hypertension who also reported depression were not at higher risk of CVD incidence than those who were not depressed. Nevertheless, in the age, sex, and health adjusted model depression was found to be an effect modifier in the relationship between diabetes/hypertension and CVD mortality (Table 3): people with diabetes/hypertension who were also depressed were 1.35 more likely to die of CVD than those without diabetes/hypertension who were also depressed (p-value = 0.037 95%CI: 1.02;1.78). In the fully adjusted, the association was no longer significant.

### The role of obesity

In the age and sex adjusted model there was evidence that obese people were at higher risk of CVD incidence (Table 2) than people with normal weight (SHR 1.23 95%CI:1.04; 1.45), however the effect disappeared in the full model and there was no evidence for an effect modification between obesity and diabetes/hypertension. Obesity was not significantly associated with higher risk of CVD mortality (Table 3) in both the age and sex-adjusted models and the fully adjusted models. We did not find evidence for obesity being an effect modifier either.

### Combined and individual effects of hypertension and diabetes on CVD risk and mortality

In Table 4, we show the associations between a categorical variable of the exposure, indicating whether respondents reported diabetes, hypertension, both or neither of the diseases. The left part of the table shows the fully adjusted SHR and corresponding 95% CI for CVD incidence, and the right part of the table shows the fully adjusted HR and 95% CI for CVD mortality. After accounting for all confounding factors, having both, diabetes and hypertension increases the risk of CVD incidence and mortality by over 30%. Having hypertension only is associated with a 1.62 higher risk of CVD incidence and 1.25 higher risk of CVD mortality, accounting for all other factors. Having only diabetes is associated with a risk of CVD incidence ranging from 0.82 to 1.90, and a risk of CVD mortality ranging from 0.61 to 1.61 (after accounting for all other variables), the non-significant association is most likely due to low power in this group.

**Table 4:** SHR and HR and 95%Confidence Intervals for CVD incidence and mortality according to diabetes/hypertension status.

	CVD incidence	CVD mortality
	SHR (95%CI)	HR (95%CI)
**Diabetes/Hypertension status**		
None	**Ref**	**Ref**
Diabetes	1.25 (0.82; 1.90)	0.99 (0.61; 1.61)
Hypertension	1.62 (1.42; 1.85)	1.25 (1.04; 1.50)
Both	1.37 (1.01; 1.85)	1.32 (0.96; 1.81)
Age	1.02 (1.01; 1.03)	1.03 (1.01; 1.04)
Female	0.83 (0.73; 0.94)	0.74 (0.62; 0.90)
Non-white ethnicity	0.49 (0.27; 0.89)	0.72 (0.33; 1.55)
Not cohabiting with a partner	1.07 (0.92; 1.23)	1.23 (1.03; 1.47)
Low education	1.02 (0.89; 1.17)	0.78 (0.65; 0.94)
Not in paid work	1.10 (0.95; 1.28)	1.02 (0.81; 1.28)
Medium wealth	1.15 (0.98; 1.35)	1.26 (0.98; 1.61)
Poorest wealth	1.02 (0.85; 1.22)	1.37 (1.06; 1.76)
Depressed	1.32 (1.13; 1.55)	0.77 (0.61; 0.96)
1+ Chronic conditions	1.11 (0.97; 1.27)	0.97 (0.80; 1.18)
Fair/poor SRH	1.02 (0.86; 1.22)	1.35 (1.08; 1.69)
Limiting Longstanding illness	0.99 (0.83; 1.18)	0.99 (0.79; 1.24)
Daily alcohol consumption	1.01 (0.88; 1.16)	1.13 (0.93; 1.38)
Current smoker	0.98 (0.90; 1.07)	0.96 (0.84; 1.09)
Physically inactive	1.07 (0.89; 1.29)	1.30 (1.04; 1.61)
Overweight	1.03 (0.88; 1.20)	1.06 (0.86; 1.31)
Obese	1.14 (0.96; 1.35)	1.13 (0.89; 1.42)
Memory CF	1.02 (1.00; 1.04)	1.00 (0.97; 1.02)
Executive CF (animal naming)	1.03 (1.01; 1.04)	0.97 (0.95; 0.99)
1+ ADLs	1.17 (0.97; 1.41)	0.95 (0.75; 1.20)
1+ IADLs	0.92 (0.76; 1.11)	1.08 (0.84; 1.38)
1+ Mobility	1.28 (1.10; 1.48)	1.09 (0.88; 1.35)

Note. 25 CVD events in people with diabetes, 469 in people with hypertension, 50 in people with both and 489 in people with neither. 19 CVD deaths in people with diabetes, 229 in people with hypertension, 50 in people with both and 258 in people with neither.

### Sensitivity analyses

In the Supporting Information, we showed the relationship between diabetes/hypertension and incident CVD and stroke separately (S1 and S2 Figs). The results are similar to those found above, showing that people with diabetes/hypertension are more likely to develop CVD than those without. The magnitude of the associations of SHRs for stroke are much stronger, showing that people with diabetes/hypertension are more than twice as likely to report incidence stroke than those without. Although all results are statistically significant, the confidence intervals of SHRs for stroke are wider due to the small number of incidence cases (N = 300).

## Discussion

Using a large nationally representative sample of older people living in private households in England we showed that people with diabetes and /or hypertension are more likely to develop CVD and also at higher risk of CVD mortality, compared to people that remain free of diabetes/hypertension. These associations were not explained by factors that are common to both diabetes/hypertension and CVD incidence and mortality, including cognitive and physical function. We also observed that each disease on its own is associated with higher risk of CVD mortality and incidence, and that the combined effect of having both diabetes and hypertension yielded to a 30% increase in CVD outcomes. Our findings are line with previous studies showing that hypertension and/or diabetes are related to higher risk of CVD incidence and mortality [3,5,7,12]. Furthermore, our results showing that both diabetes and hypertension increase the risk of CVD outcomes in is line with previous studies showing their synergistic role in elevating cardiovascular disease risk [31] and a graded increase in the risk of incident heart failure with the increasing number of risk factors such as hypertension, obesity, and diabetes [32].

We also explored the role of depression and obesity in these relationships. Contrary to our hypothesis, there was no evidence of an effect modification of depression once all the other variables were accounted for. Contrary to our results, several studies reported increased risk of cardiovascular events among people with diabetes who also reported depression [16,26,27], it is possible that by combining diabetes and hypertension cases we were not able to establish the link with CVD events, however this was necessary due to the small number of people with only diabetes in our sample. It has been also suggested that diabetes and hypertension share similar underlying molecular mechanisms, such as oxidative stress, inflammation and fibrosis, which lead to vascular complications [33].

Furthermore, when accounting for all the other variables, obesity was not associated with higher risk of CVD incidence and mortality and we did not find evidence for obesity being an effect modifier either. The nonsignificant effect-modifying role of obesity on the relationship between diabetes/hypertension and CVD incidence and mortality in our study may suggest that its effect on CVD incidence and mortality may be more indirect through its effect on CVD risk factors, including diabetes and hypertension. It has also been suggested that obesity is a heterogeneous condition in which individuals with similar BMIs may have different metabolic and CVD risk profiles [34].

However, depression was significantly associated with higher risk of CVD incidence and mortality over and above diabetes/hypertension status. This is consistent with a previous finding from a pooled analysis of 563,255 participants of a significant association between depressive symptoms and CVD incidence over and above several CVD risk factors of CVD, such as systolic blood pressure and diabetes [35].

The significant association between depression and CVD incidence and mortality may be partly explained in terms of adherence to medical recommendations. Diagnoses of hypertension or diabetes require considerable lifestyle changes and adherence to medical recommendations such as healthy diet, exercise, and smoking cessation. However, depression may interfere with patients’ ability of self-care[36] or motivation to adhere to taking medications and health behaviours. For instance, higher rates of smoking and lower levels of physical activity were observed in individuals with hypertension and common mental disorder [18].

Another possible explanation for the impact of depression in CVD incidence and mortality may be related to the depression-inflammation association. For example, in a study with 667 outpatients with CHD, greater depressive symptoms were associated with higher subsequent levels of IL-6 and CRP, and persistent depression had a greater effect on inflammation than a single episode of depression. However, this association was no longer significant after adjusting for physical inactivity, smoking, and higher BMI related to depression, suggesting a mediating role of these health behaviours in depression-inflammation association [37]. Furthermore, a previous review identified 24 cardiovascular and metabolic disease genes implicated in depression and suggested that the link between depression and cardiovascular and metabolic disease could be explained by these common genes and shared biological pathways [38].

Taken together, a complex interplay of diabetes/hypertension, depression, and obesity in CVD incidence and mortality, involving many different biological pathways, is not fully understood and warrants further investigation.

Current results suggest that in order to effectively reduce the risk of CVD incidence and mortality among older people, treatment as well as management of hypertension and diabetes are needed. Comprehensive measures such as blood sugar control, weight management and early diabetes detection and interventions can effectively help in reducing the CVD burden in older people. Management of hypertension has also been shown to reduce the incidence of stroke, especially among people with diabetes [39]. But most importantly, there is evidence that, irrespective of starting blood pressure, effectively lowering levels of blood pressure, significantly reduces the risk of CVD and stroke incidence and mortality, including all-cause mortality [40]. Previous studies have also suggested lifestyle interventions that promote a healthy lifestyle, such as regular physical activity and exercise, as an evidence-based intervention not only for the prevention and adjuvant treatment of hypertension [41], but also for the prevention of CVD in patients with diabetes [42]. Lifestyle interventions can have multiple benefits, from lowering blood pressure to improving the effectiveness of some blood pressure medications, to promoting aspects of metabolic and vascular health [43].

Furthermore, current results suggest a potential beneficial effect of treating depression to attenuate the risk of CVD incidence and mortality. In a short-term longitudinal study with inpatients in cardiac units, improvement of depressive symptoms showed a significant and independent positive association with medication adherence and secondary prevention behaviours (e.g., adherence to a diet, exercises, stress management, and medication) throughout a 6-month period, which may reduce further events or the risk of mortality [44]. Moreover, given that patients have multiple health conditions that need to be addressed simultaneously, a collaborative approach aiming at both patient and physicians appears to be effective. For instance, a team-based care management program for 214 patients with diabetes, heart disease and comorbid depression was associated with a more frequent and timely treatment adjustment for antidepressants, insulin and antihypertensive medications by physicians and more frequent patients’ self-monitoring blood pressure and glucose, leading to improved control of diabetes, depression, and heart disease [45]. Untreated depression increased secondary health care utilization and related costs in patients with hypertension and/or diabetes, suggesting that treatment of depression is beneficial in terms of reducing the health care costs [46].

Strengths of this study include a large longitudinal sample representative of older people living in private households in England. Mortality was ascertained through linkage to mortality records and therefore objectively measured. We accounted for a wide range of important factors in the association between diabetes/hypertension and CVD incidence and mortality, including cognitive and physical function. The use of competing risk analysis allowed accounting for mortality and drop-out as competing events in the CVD incidence models. A possible limitation of the study is the self-reported measures of chronic conditions. Objective measures, such as clinical diagnoses in medical records, would complement this limitation. However, questions about chronic conditions were worded to reduce sensitivity in both surveys (“Has a doctor ever told you that you have …”) [47]. Comparisons of self-reports of chronic conditions with medical records found acceptable levels of agreement [48]. For cancer, it has also been shown that self-report cancer diagnosis is sufficiently accurate for specific cancer sites [49].

## Conclusions

We have shown that diabetes and/or hypertension are related to higher risks of CVD incidence and mortality among older people. As the number of older people in the population is increasing, the CVD burden is also set to increase, posing challenges to health care systems. These challenges will be even more exacerbated due to the COVID-19 pandemic in which delay in diagnoses and treatments for chronic conditions occurred.

## Supporting information

S1 Fig Subdistribution hazard ratios and 95%confidence intervals for incident CHD among people with Hypertension/Diabetes compared to people without. 479 CHD events among people with diabetes/hypertension and 454 among those without.
(TIF)

S2 Fig Subdistribution hazard ratios and 95%confidence intervals for incident Stroke among people with Hypertension/Diabetes compared to people without. 180 stroke events among people with diabetes/hypertension and 120 among those without.
(TIF)

## References

[1] Collaborators; GBoDS. Global, regional, and national incidence, prevalence, and years lived with disability for 301 acute and chronic diseases and injuries in 188 countries, 1990–2013: a systematic analysis for the Global Burden of Disease Study 2013. The Lancet. 2015;386:7. doi: 10.1016/S0140-6736(15)60692-4 26063472 PMC4561509

[2] Collaborators; GDaI. Global burden of 369 diseases and injuries in 204 countries and territories, 1990–2019: a systematic analysis for the Global Burden of Disease Study 2019. The Lancet. 2020;396:19. doi: 10.1016/S0140-6736(20)30925-9 33069326 PMC7567026

[3] Chowdhury MZI, Yeasmin F, Rabi DM, Ronksley PE, Turin TC. Predicting the risk of stroke among patients with type 2 diabetes: a systematic review and meta-analysis of C-statistics. BMJ Open. 2019;9(8):e025579. Epub 2019/09/02. doi: 10.1136/bmjopen-2018-025579 31473609 PMC6719765

[4] Wilhelmsen L. Risks of untreated hypertension. A discussion. Hypertension. 1989;13(5 Suppl):I33–5. Epub 1989/05/01. doi: 10.1161/01.hyp.13.5_suppl.i33 2490826

[5] Hornsten C, Weidung B, Littbrand H, Carlberg B, Nordstrom P, Lovheim H, et al. High blood pressure as a risk factor for incident stroke among very old people: a population-based cohort study. J Hypertens. 2016;34(10):2059–65. Epub 2016/07/20. doi: 10.1097/HJH.0000000000001048 27434102 PMC5398900

[6] Dal Canto E, Ceriello A, Ryden L, Ferrini M, Hansen TB, Schnell O, et al. Diabetes as a cardiovascular risk factor: An overview of global trends of macro and micro vascular complications. Eur J Prev Cardiol. 2019;26(2_suppl):25–32. Epub 2019/11/15. doi: 10.1177/2047487319878371 31722562

[7] Afghahi H, Svensson MK, Pirouzifard M, Eliasson B, Svensson AM. Blood pressure level and risk of major cardiovascular events and all-cause of mortality in patients with type 2 diabetes and renal impairment: an observational study from the Swedish National Diabetes Register. Diabetologia. 2015;58(6):1203–11. Epub 2015/03/17. doi: 10.1007/s00125-015-3548-1 25773403

[8] Yazdanyar A, Newman AB. The burden of cardiovascular disease in the elderly: morbidity, mortality, and costs. Clin Geriatr Med. 2009;25(4):563–77, vii. Epub 2009/12/01. doi: 10.1016/j.cger.2009.07.007 19944261 PMC2797320

[9] Mills KT, Bundy JD, Kelly TN, Reed JE, Kearney PM, Reynolds K, et al. Global Disparities of Hypertension Prevalence and Control: A Systematic Analysis of Population-Based Studies From 90 Countries. Circulation. 2016;134(6):441–50. Epub 2016/08/10. doi: 10.1161/CIRCULATIONAHA.115.018912 27502908 PMC4979614

[10] Forouzanfar MH, Liu P, Roth GA, Ng M, Biryukov S, Marczak L, et al. Global Burden of Hypertension and Systolic Blood Pressure of at Least 110 to 115 mm Hg, 1990–2015. JAMA. 2017;317(2):165–82. Epub 2017/01/18. doi: 10.1001/jama.2016.19043 28097354

[11] Dall TM, Yang W, Halder P, Pang B, Massoudi M, Wintfeld N, et al. The economic burden of elevated blood glucose levels in 2012: diagnosed and undiagnosed diabetes, gestational diabetes mellitus, and prediabetes. Diabetes Care. 2014;37(12):3172–9. Epub 2014/11/22. doi: 10.2337/dc14-1036 25414388

[12] Gregg EW, Cheng YJ, Srinivasan M, Lin J, Geiss LS, Albright AL, et al. Trends in cause-specific mortality among adults with and without diagnosed diabetes in the USA: an epidemiological analysis of linked national survey and vital statistics data. Lancet. 2018;391(10138):2430–40. Epub 2018/05/23. doi: 10.1016/S0140-6736(18)30314-3 29784146

[13] Zhang L, Yang H, Yang P. The Correlation between Type 2 Diabetes Mellitus and Cardiovascular Disease Risk Factors in the Elderly. Appl Bionics Biomech. 2022;2022:4154426. Epub 2022/02/02. doi: 10.1155/2022/4154426 . 35103075 PMC8800622

[14] England; THSf. Diabetes. 2018.

[15] Brunner EJ, Shipley MJ, Britton AR, Stansfeld SA, Heuschmann PU, Rudd AG, et al. Depressive disorder, coronary heart disease, and stroke: dose—response and reverse causation effects in the Whitehall II cohort study. European journal of preventive cardiology. 2014;21(3):340–6. doi: 10.1177/2047487314520785 24491401

[16] Inoue K, Beekley J, Goto A, Jeon CY, Ritz BR. Depression and cardiovascular disease events among patients with type 2 diabetes: A systematic review and meta-analysis with bias analysis. J Diabetes Complications. 2020;34(12):107710. Epub 2020/09/15. doi: 10.1016/j.jdiacomp.2020.107710 32921574 PMC7467011

[17] Pan A, Sun Q, Okereke OI, Rexrode KM, Hu FB. Depression and risk of stroke morbidity and mortality: a meta-analysis and systematic review. Jama. 2011;306(11):1241–9. doi: 10.1001/jama.2011.1282 21934057 PMC3242806

[18] Hamer M, Batty GD, Stamatakis E, Kivimaki M. The combined influence of hypertension and common mental disorder on all-cause and cardiovascular disease mortality. Journal of hypertension. 2010;28(12):2401–6. doi: 10.1097/HJH.0b013e32833e9d7c 20724937

[19] Lloyd CE, Nouwen A, Sartorius N, Ahmed HU, Alvarez A, Bahendeka S, et al. Prevalence and correlates of depressive disorders in people with Type 2 diabetes: results from the International Prevalence and Treatment of Diabetes and Depression (INTERPRET-DD) study, a collaborative study carried out in 14 countries. Diabet Med. 2018;35(6):760–9. Epub 2018/02/27. doi: 10.1111/dme.13611 29478265

[20] Sartorius N. Depression and diabetes. Dialogues Clin Neurosci. 2018;20(1):47–52. Epub 2018/06/28. doi: 10.31887/DCNS.2018.20.1/nsartorius . 29946211 PMC6016052

[21] Li Z, Li Y, Chen L, Chen P, Hu Y. Prevalence of Depression in Patients With Hypertension: A Systematic Review and Meta-Analysis. Medicine (Baltimore). 2015;94(31):e1317. Epub 2015/08/08. doi: 10.1097/MD.0000000000001317 26252317 PMC4616591

[22] Hirani V, Zaninotto P, Primatesta P. Generalised and abdominal obesity and risk of diabetes, hypertension and hypertension-diabetes co-morbidity in England. Public Health Nutr. 2008;11(5):521–7. Epub 2007/09/05. doi: 10.1017/S1368980007000845 17767799

[23] Demakakos P, Pierce MB, Hardy R. Depressive symptoms and risk of type 2 diabetes in a national sample of middle-aged and older adults: the English longitudinal study of aging. Diabetes Care. 2010;33(4):792–7. Epub 2010/01/21. doi: 10.2337/dc09-1663 20086253 PMC2845029

[24] Guh DP, Zhang W, Bansback N, Amarsi Z, Birmingham CL, Anis AH. The incidence of co-morbidities related to obesity and overweight: a systematic review and meta-analysis. BMC Public Health. 2009;9:88. Epub 2009/03/27. doi: 10.1186/1471-2458-9-88 19320986 PMC2667420

[25] Hare DL, Toukhsati SR, Johansson P, Jaarsma T. Depression and cardiovascular disease: a clinical review. Eur Heart J. 2014;35(21):1365–72. Epub 2013/11/28. doi: 10.1093/eurheartj/eht462 24282187

[26] Hackett RA, Steptoe A. Psychosocial Factors in Diabetes and Cardiovascular Risk. Curr Cardiol Rep. 2016;18(10):95. Epub 2016/08/28. doi: 10.1007/s11886-016-0771-4 27566328 PMC5002050

[27] Jung I, Kwon H, Park SE, Han KD, Park YG, Kim YH, et al. Increased Risk of Cardiovascular Disease and Mortality in Patients with Diabetes and Coexisting Depression: A Nationwide Population-Based Cohort Study. Diabetes Metab J. 2021;45(3):379–89. Epub 2020/12/11. doi: 10.4093/dmj.2020.0008 33297602 PMC8164944

[28] Steptoe A, Breeze E, Banks J, Nazroo J. Cohort profile: the English longitudinal study of ageing. Int J Epidemiol. 2013;42(6):1640–8. Epub 2012/11/13. doi: 10.1093/ije/dys168 23143611 PMC3900867

[29] Zaninotto P, Huang Y-T, Di Gessa G, Abell J, Lassale C, Steptoe A. Polypharmacy is a risk factor for hospital admission due to a fall: evidence from the English Longitudinal Study of Ageing. BMC Public Health. 2020;20:1–7. 33243195 10.1186/s12889-020-09920-x PMC7690163

[30] Fine JPG R. J.;. A proportional hazards model for the subdistribution of a competing risk. Journal of the American Statistical Association. 1999;45(3):11.

[31] Balogun WO, Salako BL. Co-occurrence of diabetes and hypertension: pattern and factors associated with order of diagnosis among nigerians. Ann Ib Postgrad Med. 2011;9(2):89–93. Epub 2011/12/01. 25161490 PMC4111031

[32] Ahmad FS, Ning H, Rich JD, Yancy CW, Lloyd-Jones DM, Wilkins JT. Hypertension, obesity, diabetes, and heart failure—free survival: the cardiovascular disease lifetime risk pooling project. JACC: Heart Failure. 2016;4(12):911–9. 27908389 10.1016/j.jchf.2016.08.001 PMC5582802

[33] Petrie JR, Guzik TJ, Touyz RM. Diabetes, hypertension, and cardiovascular disease: clinical insights and vascular mechanisms. Canadian Journal of Cardiology. 2018;34(5):575–84. doi: 10.1016/j.cjca.2017.12.005 29459239 PMC5953551

[34] Powell-Wiley TM, Poirier P, Burke LE, Després J-P, Gordon-Larsen P, Lavie CJ, et al. Obesity and cardiovascular disease: a scientific statement from the American Heart Association. Circulation. 2021;143(21):e984–e1010. doi: 10.1161/CIR.0000000000000973 33882682 PMC8493650

[35] Harshfield EL, Pennells L, Schwartz JE, Willeit P, Kaptoge S, Bell S, et al. Association between depressive symptoms and incident cardiovascular diseases. Jama. 2020;324(23):2396–405. doi: 10.1001/jama.2020.23068 33320224 PMC7739139

[36] Ducat L, Philipson LH, Anderson BJ. The mental health comorbidities of diabetes. JAMA. 2014;312(7):691–2. Epub 2014/07/11. doi: 10.1001/jama.2014.8040 25010529 PMC4439400

[37] Duivis HE, de Jonge P, Penninx BW, Na BY, Cohen BE, Whooley MA. Depressive symptoms, health behaviors, and subsequent inflammation in patients with coronary heart disease: prospective findings from the heart and soul study. Am J Psychiatry. 2011;168(9):913–20. Epub 2011/07/05. doi: 10.1176/appi.ajp.2011.10081163 21724664

[38] Amare AT, Schubert KO, Klingler-Hoffmann M, Cohen-Woods S, Baune BT. The genetic overlap between mood disorders and cardiometabolic diseases: a systematic review of genome wide and candidate gene studies. Translational psychiatry. 2017;7(1):e1007-e. 28117839 10.1038/tp.2016.261 PMC5545727

[39] Hewitt J, Castilla Guerra L, Fernandez-Moreno Mdel C, Sierra C. Diabetes and stroke prevention: a review. Stroke Res Treat. 2012;2012:673187. Epub 2013/01/18. doi: 10.1155/2012/673187 23326760 PMC3543806

[40] Ettehad D, Emdin CA, Kiran A, Anderson SG, Callender T, Emberson J, et al. Blood pressure lowering for prevention of cardiovascular disease and death: a systematic review and meta-analysis. Lancet. 2016;387(10022):957–67. Epub 2016/01/03. doi: 10.1016/S0140-6736(15)01225-8 26724178

[41] Valenzuela PL, Carrera-Bastos P, Gálvez BG, Ruiz-Hurtado G, Ordovas JM, Ruilope LM, et al. Lifestyle interventions for the prevention and treatment of hypertension. Nature Reviews Cardiology. 2021;18(4):251–75. doi: 10.1038/s41569-020-00437-9 33037326

[42] Chen L, Pei J-H, Kuang J, Chen H-M, Chen Z, Li Z-W, et al. Effect of lifestyle intervention in patients with type 2 diabetes: a meta-analysis. Metabolism. 2015;64(2):338–47. doi: 10.1016/j.metabol.2014.10.018 25467842

[43] Association AD. Cardiovascular disease and risk management: standards of medical care in diabetes-2020. Diabetes Care. 2020;43:S111–34. 31862753 10.2337/dc20-S010

[44] Bauer LK, Caro MA, Beach SR, Mastromauro CA, Lenihan E, Januzzi JL, et al. Effects of depression and anxiety improvement on adherence to medication and health behaviors in recently hospitalized cardiac patients. The American journal of cardiology. 2012;109(9):1266–71. doi: 10.1016/j.amjcard.2011.12.017 22325974

[45] Lin EH, Von Korff M, Ciechanowski P, Peterson D, Ludman EJ, Rutter CM, et al. Treatment adjustment and medication adherence for complex patients with diabetes, heart disease, and depression: a randomized controlled trial. The Annals of Family Medicine. 2012;10(1):6–14. doi: 10.1370/afm.1343 22230825 PMC3262469

[46] Pálinkás A, Sándor J, Papp M, Kőrösi L, Falusi Z, Pál L, et al. Associations between untreated depression and secondary health care utilization in patients with hypertension and/or diabetes. Social Psychiatry and Psychiatric Epidemiology. 2019;54(2):255–76. doi: 10.1007/s00127-018-1545-7 29947863

[47] Zaninotto P, Steptoe A. Association between subjective well-being and living longer without disability or illness. JAMA Network Open. 2019;2(7):e196870-e. doi: 10.1001/jamanetworkopen.2019.6870 31290992 PMC6624816

[48] Beckett M, Weinstein M, Goldman N, Yu-Hsuan L. Do health interview surveys yield reliable data on chronic illness among older respondents? Am J Epidemiol. 2000;151(3):315–23. Epub 2000/02/12. doi: 10.1093/oxfordjournals.aje.a010208 10670557

[49] Bergmann MM, Calle EE, Mervis CA, Miracle-McMahill HL, Thun MJ, Heath CW. Validity of self-reported cancers in a prospective cohort study in comparison with data from state cancer registries. Am J Epidemiol. 1998;147(6):556–62. Epub 1998/04/01. doi: 10.1093/oxfordjournals.aje.a009487 9521182

